# Niosomes in cancer treatment: A focus on curcumin encapsulation

**DOI:** 10.1016/j.heliyon.2023.e18710

**Published:** 2023-07-26

**Authors:** Thaaranni Bashkeran, Azlina Harun Kamaruddin, Trung Xuan Ngo, Kazuma Suda, Hiroshi Umakoshi, Nozomi Watanabe, Masrina Mohd Nadzir

**Affiliations:** a School of Chemical Engineering, Universiti Sains Malaysia, Engineering Campus, 14300, Nibong Tebal, Pulau Pinang, Malaysia; b Rohto Pharmaceutical Co., Ltd., Basic Research Division, Research Village Kyoto, 6-5-4 Kunimidai, Kizugawa, Kyoto, 619-0216, Japan; c Division of Chemical Engineering, Graduate School of Engineering Science, Osaka University, 1-3 Machikaneyama-cho, Toyonaka, 560-8531, Japan

**Keywords:** Niosome, Curcumin, Cancer, Formulation, Application

## Abstract

Curcumin is widely used as a therapeutic drug for cancer treatment. However, its limited absorption and rapid excretion are the major therapeutic limitations to its clinical use. Using niosomes as a curcumin delivery system is a cheap, easy, and less toxic strategy for enhancing the absorption of curcumin by cells and delaying its excretion. Thus, there is a vital need to explore curcumin niosomes to configure the curcumin to suitably serve and aid current pharmacokinetics in treatments for cancer. To date, no comprehensive review has focused on the cytotoxic effects of curcumin niosomes on malignant cells. Thus, this review provides a critical analysis of the curcumin niosomes in cancer treatment, formulations of curcumin niosomes, characterizations of curcumin niosomes, and factors influencing their performance. The findings from this review article can strongly accelerate the understanding of curcumin niosomes and pave a brighter direction towards advances in the pharmaceutical, biotechnology, and medical industries.

## Highlights

• Curcumin is a polyphenol that has been extensively studied for anticancer property.
• Incorporating curcumin into niosomes boosts its efficacy against cancer cells.
• Synthesis parameters affect the delivery profile of curcumin niosomes.
• Clinical and non-clinical applications of curcumin niosome are critically reviewed.

## Graphical abstract

**Figure:**
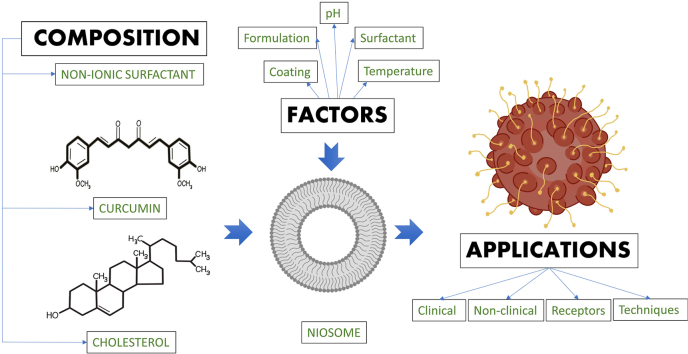

## Abbreviations

**PEG**: Polyethylene glycol
**HLB**: Hydrophilic-lipophilic balance
**MTT**: 3-(4,5-dimethylthiazol-2-yl)- 2,5-diphenyl-2H-tetrazolium bromide
**Tf**: Transferrin
**CD44**: Cluster of differentiation 44
**CD44v**: D44 variant isoforms
**EGFR**: Epidermal growth factor receptor
**VEGF**: Vascular endothelial growth receptor
**NFκB**: Nuclear Factor Kappa B
**IκB**: inhibitor of NFκB

## 1 Introduction

*Curcuma longa* L. is commonly known as turmeric. This plant is a member of the ginger family (Zingiberaceae) endemic to southwest India and produces a brilliant yellow spice from its rhizomes. The yellow color of turmeric is due to the three primary curcuminoids (i.e., demethoxycurcumin, bis-demethoxycurcumin, and diferuloylmethane (curcumin)) found in rhizomes [1]. Dried *C. longa* rhizome powder contains an average curcuminoid composition of 75% curcumin, 10%–25% demethoxycurcumin, and 5% bis-demethoxycurcumin. Over the last few decades, interest in and studies on the active compounds of turmeric, especially curcumin, have increased. In 2017, the global market for curcumin reached 52.45 million USD, and is expected to reach 104.19 USD by the year 2025. The annual compound growth rate was 8.9% from 2018 to 2025 [2]. Curcumin is a compound with health properties that make it an ideal constituent in the food and medical industries. The market demand for the use of curcumin is expected to evolve as consumers' knowledge of curcumin continues to expand [2]. This is undeniable owing to the benefits of curcumin, such as its affordability, safety, and efficacy as an agent compared to other curcuminoids [3,4]. The anti-cancer properties of curcumin aid in alleviating cancer's complex and interconnected network of transcription factors, which encompass crucial regulators such as oncogenes [5], tumor suppressor genes [6], and genes associated with aberrant cell proliferation and survival pathways. By exerting its effects on these intricate molecular mechanisms, curcumin presents itself as an enticing alternative for cancer treatments, capable of specifically targeting and modulating the intricate web of transcriptional factors that contribute to cancer development and progression [7].

Cancer is one of the leading causes of mortality worldwide, accounting for nearly six million deaths each year [8]. Cancer is caused by both genetic and epigenetic alterations. Consequently, apoptosis, metastasis, angiogenesis, and unlimited cell proliferation can occur. Innumerable and paramount medications have been developed from scientific examinations of the plants used in many forms of ethnic medicines, including taxol, camptothecin, vincristine, and vinblastine [9]. Curcumin has been shown to have anti-cancer properties (whether used alone or in conjunction with traditional chemotherapy medications) for treating cancer such as breast cancer and ovarian cancer [10,11]. According to *in vitro* and *in vivo* studies, curcumin inhibits carcinogenesis by altering two basic processes: angiogenesis and tumor development [12].

Despite the well-received pharmacological qualities of curcumin, its medicinal use is hampered by its limited water solubility at acidic and physiological pH levels, poor stability, short half-life, and degradability under alkaline conditions. Curcumin's bioavailability is further bound by its low absorption and rapid metabolism [13]. These drawbacks can be resolved by encapsulating curcumin in an established drug delivery system. A drug delivery system can be easily described as a system for directing a curative and remedial drug to a targeted site in the human body. Hence, the system should be monitored and controlled to achieve a desirable duration and drug release profile [14]. Multiple diversified drug delivery systems have been established over the years and forced prodigious advancement. One of the leading superlative drug delivery systems is the niosome.

Niosomes are bilayer-structured non-ionic surfactant vesicles employed to transport a variety of pharmacological components, such as hormones, antigens, therapeutic drugs, genes, and peptides [15]. Niosomes are made up of non-ionic surfactants with a preeminent function of acting as the vesicular layer of the niosomes, whereas the addition of compounds such as cholesterol ameliorates the stiffness of the vesicle bilayer. This pronounced carrier system protects drug molecules against immunological and pharmacological actions that induce degradation and inactivation. The cell membrane-like components in niosomes promote enhanced permeation through the cellular membrane via osmosis and significant biocompatibility. Furthermore, niosomes outperform other drug delivery systems in terms of production costs and storage of the chemicals at room temperature [16].

In recent years, niosomes have gained popularity as drug delivery systems, especially for medicines with inconsiderable stability, restricted solubility, or rapid breakdown [17]. Furthermore, niosomes have shown great potential as drug delivery systems in cancer research. Despite accessible formulations and an abundance of drugs, cancer treatment remains a major challenge for researchers. One of the primary challenges in developing and curating innovative drug delivery systems for targeting cancer cells is in improving the cancer cell elimination efficiency while retaining sufficient systematic biocompatibility *in vivo* [18]*.* In the context of using niosomes as the delivery system for curcumin for cancer treatments, the influences of parameters such as the niosome formulation, pH condition, membrane surface charge, nature of the surfactant, and temperature of the dispersion medium remain unclear. As such, this review aims to improve comprehension regarding building a tailored niosomal vesicular system for cancer treatments. In addition, this review paper also addresses the aforementioned issues in curcumin niosomes. The graphical abstract effectively captures the essence of the review paper, providing a succinct summary of its content and key findings.

## 2 Cellular and molecular mechanism of curcumin against cancer cells

Curcumin has been shown to have anticancer properties whether used alone or in conjunction with traditional chemotherapy medications to treat cancer and cancer-related problems [11]. Curcumin inhibits carcinogenesis by altering two basic processes: angiogenesis and tumor development, according to *in vitro* and *in vivo* studies [12]. The idea that curcumin operates on various biochemical and molecular cascades is supported by the fact that it has wide variety of molecular targets. Up to 33 distinct proteins, including thioredoxin reductase, cyclooxygenase-2 (COX2), protein kinase C, 5-lipoxygenase (5-LOX), and tubulin, are physically bound by curcumin. Transcription factors, growth factors and their receptors, cytokines, enzymes, and genes governing cell proliferation and death are only a few of the molecular targets that this substance modulates. It has been demonstrated that curcumin prevents practically all types of tumor cells from proliferating and surviving. The manner of curcumin-induced cell death is thought to be mediated through the activation of cell death pathways as well as the inhabitation of growth and proliferation pathways [19]. Curcumin has been demonstrated to supress angiogenesis and stop the spread of cancer cells *in vivo*. The primary organelle for protein synthesis, maturation, and creation of the protein's spatial structure is the endoplasmic reticulum (ER). The balance between survival and apoptotic signals influences cell destiny to some extent, and the particular ER stressor is crucial in regulating homeostasis. Apoptotic cell death is triggered by increased stress in ER. When ER transmembrane protein factors notice a build-up of unfolded proteins, unfolded protein response is initiated in the cell [20]. The cellular and molecular mechanism of curcumin's action on cancer cells can be divided into several key pathways. Firstly, inhibition of NFκB signaling pathway where curcumin has been shown to inhibit the activity of NFκB, as a transcription factor that plays a key role in regulating inflammation and cell survival. NFκB is often overactivated in cancer cells promoting their growth. By inhibiting NFκB, curcumin can reduce cancer cell growth and induce apoptosis. Next, curcumin has been shown to modulate the activity of cell cycle regulatory proteins such as cyclin D1 and cyclin-dependent kinases that leads to cell cycle arrest [19].

## 3 Curcumin niosome

**Fig. 1:**
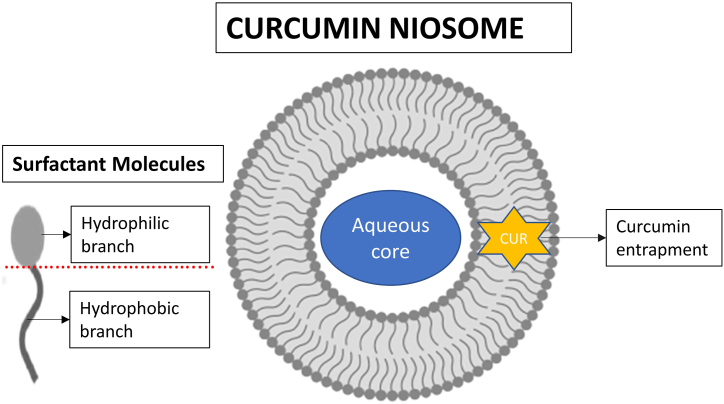
The structure of curcumin niosome consisting of surfactant molecules arranged in a bilayer structure. The aqueous core is situated in the center of the vesicle, serving as the encapsulation space for curcumin.

Niosomes are tiny (10–1000 nm), non-ionic, surface-active compounds with a bilayered structure consisting of a hydrophilic head, hydrophobic tail, and aqueous core nestled in the middle of the vesicle [14]. These vesicles comprise colloidal particles generated when non-ionic surfactants (with or without cholesterol and therapeutic moieties) self-assemble in aqueous fluids, resulting in a microscopic lamellar bilayer structure. These thermodynamically stable bilayered structures develop only when the surfactants and cholesterol are combined in the right proportions and the temperature is above the gel-liquid transition point [21,22]. The core of this bilayered construction is a hollow void. Niosomes may encapsulate both hydrophilic and hydrophobic drugs, owing to their amphiphilic nature and unique shape. Hydrophilic pharmaceuticals entrapped in niosomes can be adsorbed on the bilayer surface or entrapped in the core aqueous domain, whereas hydrophobic drugs can be partitioned within the bilayer structure [23,24]. Niosomes can encapsulate curcumin within their lipid bilayers. Owing to the hydrophobic nature of curcumin, it is generally entrapped in a non-polar area or the hydrophobic regions of the bilayer while the hydrophilic headgroups of surfactants form the outer layer of niosome. The bilayer structure of the curcumin niosome, with two distinct regions for drug entrapment, is illustrated in Fig. 1. Most of the synthesis method of niosome utilizes the self-assembly properties of the lipid components and the hydrophobic nature of curcumin to facilitate the encapsulation process. Most commonly, Fourier transform infrared spectroscopy (FTIR) is used to investigate the interactions between curcumin and niosome in the molecular level. FTIR spectra can reveal shifts in characteristics peaks, suggesting possible interactions or changes in chemical bonding [25]. When present in a solution, curcumin is primarily found in the form keto-enol tautomer with a notable intramolecular hydrogen bond in ground state. The presence of water molecules in the hydration layer surrounding the niosome plays a crucial role in regulating the photophysical processes. Niosomes can introduce desirable nanostructures that offer enhanced protection to encapsulated curcumin, shielding them from interactions with water. The presence of cholesterol molecules in the niosome membrane elevates its rigidity, leading to increased interactions between curcumin and non-ionic surfactant. Moreover, the hydroxyl groups of cholesterol can potentially form intermolecular hydrogen bonds with curcumin, which can perturb the non-radiative deactivation mechanism through excited-state intramolecular hydrogen atom transfer [26]. The incorporation of curcumin into the particles causes a slight increase in their hydrodynamic size. In a study, blank niosomes exhibited smaller particles size (182.6 nm) in relative to curcumin niosome with varying encapsulated mass of curcumin ranging from 1 mg to 3 mg (223.7 nm–235.1 nm). As the amount of curcumin encapsulated increases, the hydrodynamic size of the particles also increases. This increase is attributed to the diffusion of curcumin molecules between the surfactant tails within the bilayer of the niosomes [27]. Niosomes loaded with curcumin show a low polydispersity index indicating uniformity in size. However, the polydispersity index results also indicate that the number of drug molecules can influence the distribution of hydrodynamic sizes within the niosome population. The zeta potential values of vesicles also vary depending on the amount of curcumin loaded. With an increase in curcumin amount, more negative zeta potential values are observed [28]. This negative charge can be attributed to the inherent negative charge of curcumin itself. Curcumin is known to be susceptible to degradation caused by factors such as light, heat, oxygen and enzymatic activity [29]. When encapsulated within niosomes, curcumin is shielded from direct exposure of these degradation factors, thus increasing its stability. Also, niosome provides a hydrophobic core where curcumin can be encapsulated, reducing its exposure to water and minimizing hydrolysis. The stability of curcumin can be monitored in three different ways such as physical stability, thermal stability and chemical stability. Usually, the curcumin niosomes will be stored under specific conditions, such as refrigeration, freezer or room temperature, and periodically evaluate their stability parameters over an extended period. This method helps assess their stability under storage conditions [13]. As reported by many researchers, most of the curcumin niosome can withstand their stability at varying temperature for months [7,13,27,28,30]. Elevated temperatures can enhance the fluidity of the bilayer structure in niosomes by reducing the electrostatic repulsion between vesicles and entropic forces among the surfactant head groups. This increased fluidity may result in the leakage of curcumin, leading to decrease in vesicle size [30]. However, it is important to note that the curcumin stability in niosomes can be influence by various factors, including the composition of the niosome formulation, storage conditions, and the presence of stabilizing agent.

The desired characteristics of curcumin niosomes can be managed by modifying the vesicle formulation. The surfactants involved in the formulation of curcumin niosomes are biodegradable, biocompatible, non-immunogenic, and non-toxic. Moreover, the encapsulation of curcumin into niosomes protects it from enzyme metabolism, enhances the stability of the curcumin, improves the permeation of the curcumin through the skin, and improves its curative profile (as engendered by the detained clearance from drug circulation) [28].

There are also other types of delivery systems for curcumin, as shown in Table 1. Table 1 also presents the differences between drug delivery systems. Although niosomes are superior to some of the existing drug carriers, they have certain drawbacks. First, niosome formulations and compositions are physically unsteady. Another disadvantage is that niosomes can exhibit aggregation if the standard method of preparation is not obeyed. Thus, there is also a possible risk of curcumin hydrolysis. In addition, niosome preparation is a time-consuming procedure. Rare cases of inadequate drug loading have also been reported. At times, dissimilar surface charges may become available on the vesicle; these make it vulnerable to fusion, as opposing charges have a higher probability of being attracted to the vesicle [31].

**Table 1:** Comparison of curcumin delivery systems in terms of the composition, advantages, and disadvantages.

Drug delivery system	Composition	Advantages	Disadvantages	References
Phospholipid complexes	Phospholipids	•Flexible •Maintain strength •Improved bioavailability and absorbance of curcumin •Improved solubility of water and lipid	•Complex natural lecithin may hydrolyze and form Lys phospholipids •May lead to hemolysis	[32]
Microemulsion, self-emulsion and nanoemulsion	Surfactant	•Better solubility •Improved stability •Economical scale up	•Usage of large concentrations of surfactant and co-surfactant •Solubility capacity is limited by highly melting substances	[33]
Liposome	Phospholipids	•Biocompatibility •Ability to self-assemble •Ability to encapsulate large payloads of drugs •Ability to control and modify biological characteristics	•High production cost •Leakage of incorporated drug •Phospholipid may undergo oxidation and hydrolysis •Short half life	[34]
Polymeric micelles	Amphiphilic block copolymers	•Alleviated side effects of drugs •Easy scale up •Slows down opsonization •Prolonged circulation time	•Poor physical stability •Poor drug-loading efficiency •Insufficient cellular interaction •Smaller drug loading capacity	[35]

## 4 Comparison of niosome and liposome

Niosomes are structurally similar to liposomes and polymersomes, which are nanostructures made up of a bilayer of phospholipids or copolymers, respectively. Niosomes, on the other hand, are superior to liposomes in terms of chemical stability and cost. Niosomes are tiny lamellar structures (10–1000 nm) whose amphiphilic nature allows hydrophilic medicines to be entrapped in the core cavity and hydrophobic medications to be entrapped in the non-polar area of the bilayer [23]. This carrier system protects the drug molecule against immunological and pharmacological actions that induce degradation and inactivation. Due to their cell membrane-like components, niosomes, like liposomes, have better cellular membrane permeation via osmosis and great biocompatibility. Furthermore, niosomes outperform liposomes in terms of cheap production costs and chemical storage needs at room temperature. In recent years, niosomes have gained popularity as a drug delivery alternative to liposomes, especially for medicines with poor stability, limited solubility, or fast breakdown [17]. A research illustrated that Span 80 vesicles exhibit a more fluid and flexible membrane compared to liposomes. Unlike liposomes, the membrane surface of Span 80 vesicles can accommodate water molecules. While similarities were observed in the inner portion of the membrane Span 80 vesicles and liposomes, notable differences were noted in the outer region. These disparities encompassed the membrane's polarity, head group charge and surface mobility [36]. Table 2 shows the brief summary of the comparison between liposome and niosome.

**Table 2:** A comparative analysis of niosomes and liposomes in terms of price, charge, stability, and special conditions/methods.

Parameters	Niosome	Liposome
Price	Less expensive	More expensive
Charge	Non-ionic surfactants are neutrally charged	Phospholipids are neutrally charged
Stability	Non-ionic surfactants are utilized for stability of vesicle	Phospholipids are vulnerable to oxidative degradation
Special condition/method	Requires no special condition/method for formulation	Requires special condition/method for handling, storage and preparation of phospholipids

## 5 Synthesis method of niosome

The niosome preparation methods can be divided into three categories that comprises of passive trapping techniques, active trapping techniques and miscellaneous methods. Passive trapping techniques is utilized when drugs like curcumin is encapsulated during the devising of the curcumin niosome whereas active trapping techniques involves incorporating curcumin post niosome preparation. Miscellaneous method comprises of the heating method, emulsion method and lipid injection method [14]. The most common and generally used methods to prepare niosome are discussed below.

### 5.1 Ether injection

In this method, non-ionic surfactant and cholesterol are introduced to diethyl ether mixed with methanol and curcumin. The resulted solution is injected into hydrating phosphate buffer at a constant rate using micro syringe on a magnetic stirrer set at a temperature of 60 °C–65 °C. The difference in temperature forces vaporisation of ether that would lead a layer of niosome vesicle to form [37]. Ether injection process composing of curcumin, Span 80, diethyl ether and cholesterol can be used to synthesize proniosome. The curcumin content in the niosome was at maximal level under refrigerated storage condition followed by room temperature and high temperature. The drug content at refrigerated, room and elevated temperature condition was valued at 99.5%, 99.2% and 93%, respectively. Other than that, the incorporation yield of curcumin ranged from 82.3% to 86.8% [38].

### 5.2 Sonication

In a scintillation vial, the drug-containing aqueous phase is introduced to a combination of surfactant and cholesterol. A sonic probe is used to homogenise the mixture at 60 °C for 3 min. Small and homogeneous sized vesicles is formed [39]. Recent advances on sonication technique are the high energy probe sonication. Drug such as rifampicin and ceftriaxone are encapsulated in niosome are synthesized using this method. In the study, the size of vesicle ranged from 165 to 893 nm with polydispersity index (PDI) valued at 0.333 to 0.725 whereas the entrapment efficiency of rifampicin and ceftriaxone achieved high values that exceeds 96% [40]. In a study, niosomes synthesized using thin film hydration and ultrasonic processing were distinguished. The results exhibited that niosomes synthesized from ultrasonic method is smaller with lower PDI, this show that these niosomes are more stable and dispersed [41].

### 5.3 Thin film hydration method

This method is also known as the hand shaking method. A rotary evaporator rotating at 120 rpm is commonly utilized where surfactant and cholesterol is dissolved into a volatile organic solvent such as methanol, chloroform, ethanol, and diethyl ether. Thin film is formed on the wall of flask. The dried surfactant film is then rehydrated with aqueous phase containing curcumin at room temperature with slight agitation mainly to produce multilamellar vesicles niosome [42]. Thin film hydration method is a successful technique to encapsulate etoricoxib utilising surfactants and cholesterol. When Span 60 was used as non-ionic surfactant, the etoricoxib content was highest at 95.57% with diameter of niosome at 463.7 nm. Hence, thin film hydration method is effective for incorporating etoricoxib into niosome [43].

### 5.4 Reverse phase evaporation technique

In the reverse phase evaporation (REV) technique, cholesterol and surfactant at equal ratio were dissolved into organic solvent. Curcumin in aqueous phase is added where it results to water in oil emulsion. This double phase emulsion is sonicated via sonicator at 4–5 °C. Then, the rotary evaporator is utilized to produce gel of bigger vesicles. Phosphate buffer saline (PBS) is included to semisolid gel to be sonicated again. At lower pressure, organic solvent is eliminated. Slurry niosomal suspension is watered down using PBS. Lastly, niosome is produced after heating it over a water bath for 10 min at 60 °C [43]. Curcumin encapsulated cationic unilamellar niosomes was synthesized using REV and then was frozen in a dried condition. The PDI and the entrapment efficiency of the vesicle was 97.4 nm and 83.3%, respectively [44].

### 5.5 Microfluidisation

This method focuses on submerged jet principle. At high velocities, interaction between two fluidized streams take place in the interaction chamber packed with ice via micro channels. This solution is then sent through a loop to cool and dissipate the heat created during the process. Additionally, a customary gateway is set up so that the energy delivered to the system stays within the niosome formation zone [14]. Tenofovir encapsulated niosome was prepared using microfluidisation technique where Span 20, Span 40, Span 60 and cholesterol was incorporated in the composition. The release profile of tenofovir is highly influenced by the formulations compared to physical condition. This proves and justifies the functionality of microfludisation technique and elevates the ease of the scaling up process of tenofovir as vesicles with smaller diameter is produced [45].

### 5.6 Multiple membrane extrusion method

This method focuses on the usage of membrane that is arranged in a series manner to undergo extrusion. A mixture containing surfactant, cholesterol and diacetyl phosphate is dissolved in organic solvent such as chloroform. The emanated solution is evaporated to produce a thin layer. Aqueous solution consists of curcumin hydrates the thin surfactant film formed. Then, this suspension is extruded via polycarbonate membranes that are situated in series [46]. There are studies where multiple membrane extrusion method is done followed by a thin film hydration techniques. For instance, doxycycline hyclate incorporated niosome was prepared using thin film hydration method followed by multiple membrane extrusion technique. The particle size of niosome synthesized by this method ranged from 117 to 209 nm. As the content of cholesterol increases from 30 mol% to 50 mol%, the entrapment efficiency decreases from 59% to 45% [47].

## 6 Types of curcumin niosomes

**Fig. 2:**
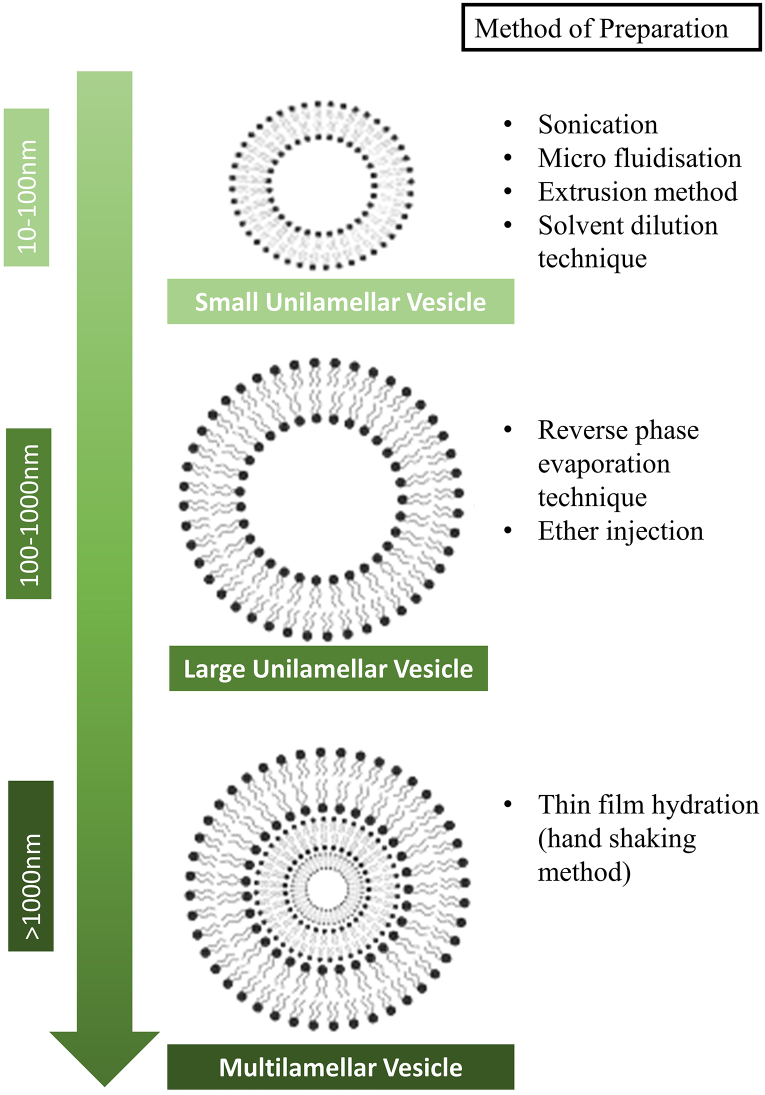
Schematic diagram of types of niosomes and representation of their distinctive structures, including small unilamellar vesicles, large unilamellar vesicles and multilamellar vesicles and their respective method of preparation.

Curcumin niosomes (or niosomes in general) can be classified based on the distribution and size of the drug delivery vesicles, number of bilayers present in the vesicle, entrapment efficiency, and permeability. The two main factors for categorizing niosomes are the vesicle size and method of preparation. The size of curcumin niosomes ranges from 10 to 1000 nm. The suitable diameter for a biomedical application can vary depending on the specific use and the type of material being used. In general, the diameter should be large enough to allow for the necessary flow or function, but not so large as to cause damage or interfere with the surrounding tissue. To optimize the diameter for a specific biomedical application, it is important to consider the specific requirements and constraints of the application. This may involve factors such as desired flow rate, the properties of the material being used, the surrounding tissue and any potential risks or complications. In addition, computational modelling and simulation can be used to help optimize the design of biomedical drug delivery system and identify the most suitable diameter for a given application. Given that the vesicle size has a significant impact on the vesicle biodistribution, it is frequently necessary to incorporate a size reduction step after the first hydration stage in the niosome synthesis process [48]. In addition, different preparation methods allow for the formation of niosomes with different sizes. In general, there are three types of niosomes: small unilamellar vesicles, multilamellar vesicles, and large unilamellar vesicles. Fig. 2 shows a schematic diagram of the three types of curcumin niosomes.

The formation of any type of curcumin niosome is highly dependent on the method of preparation. The sizes of small unilamellar vesicles range from approximately 10 to 100 nm. Small unilamellar vesicles can be formed by sonication, in which large unilamellar vesicles are converted to small unilamellar vesicles under appropriate conditions (60 °C, 1 h). Moreover, micro-fluidization, an extrusion method where French press extrusion electrostatic stabilization is utilized, and a solvent dilution technique can also be used to produce small unilamellar vesicles. These procedures are known to be time-efficient [31].

Multilamellar vesicles can be easily differentiated from other types of vesicles, as they contain only a few layers (known as bilayers) independently encircling the aqueous lipid receptacle. Multilamellar vesicle niosomes are mostly utilized for drug encapsulation, especially for lipidic drugs. The mean diameter of multilamellar vesicles is between 0.5 and 10 μm. Compared to small unilamellar vesicles and large unilamellar vesicles, multilamellar vesicle niosomes exhibit better stability over an extended period. The thin film hydration method, also known as the hand-shaking method, is widely used to produce multilamellar vesicle niosomes [14]. This method consists of a simple procedure that improves stability upon retaining the extended phases [14].

The large unilamellar vesicles is a large-diameter membrane consisting of a single bilayer. The average size of the vesicles range from100 to 1000 nm. The large unilamellar vesicles can carry a heavier content of the aqueous phase and lipidic compounds, as the large unilamellar vesicles permits a higher entrapment quantity. Reverse-phase evaporation and ether injection are two methods broadly employed to form large unilamellar vesicles [39]. The advantages of synthesizing large unilamellar vesicles include a high proportion of aqueous-to-lipid sections and forgeable drug release profile rate [39].

## 7 Factors affecting the performance of curcumin niosome

### 7.1 Formulation of curcumin niosome

The building blocks of curcumin niosomes comprise a proper ratio of cholesterol and ionic surfactants. Cholesterol has sage reverberation in terms of the curcumin niosome formulation. In studies of cationic polyethylene glycol (PEG)-ylated normal formulations, an increase in cholesterol content from 10% to 30% showed a gradual increase in the entrapment of curcumin and in the mean diameter of the curcumin niosomes. However, there was a slight drop in the percentage of curcumin released within 12 h [49]. In addition, the ratio of Tween 60 to Tween 80 is a prominent factor influencing the encapsulation efficiency, curcumin release rate, and size of curcumin niosomes; it is particularly significant to their ability to cross the blood-brain barrier and accumulate in the target organs [50]. In one study at a constant curcumin concentration, cationic niosomal formulations containing Tween 60 and Tween 80, cholesterol, and dioleyl-3-trimethylammonium propane delivered enhanced results in terms of the drug release profile and encapsulation efficiency of the curcumin. In a different study, hyaluronan was incorporated into a polymeric niosomal structure synthesized via a thin film hydration medium to boost the curcumin efficiency. The diameter of hyaluronan-containing niosomes was 249.83 ± 6.38 nm, and the curcumin entrapment was 98.28 ± 0.278% (w/w). Furthermore, the niosome-containing hyaluronan had a spherical form, indicating a stable niosome [51]. Notably, a larger amount of surfactant is required for transdermal administration relative to intravenous administration to overcome the stratum corneum barrier and preserve deformability for penetration into target cancer cells. To support this, a conjugate-designed hydrochloric acid niosome using hydrochloric acid esterified with monostearin has shown superior performance in endocytosis to mouse breast tumor cells (4T1) compared to a chitosan (CH) control nanoparticle. The 4T1 cell tumor development and metastatic dissemination in mice closely resemble those in human breast cancer. The increase in cytotoxicity achieved by hydrochloric acid niosomes was up to 3% m/v owing to the Tween 80 and Span 20 surfactants used during the synthesis of the niosomes. *In vivo* fluorescence monitoring depicted direct proof of cellular absorption, where the fluorescence intensities of 4T1 cells treated with hydrochloric acid niosome for 0.5 h were higher than that of the CH nanoparticle [52].

### 7.2 pH condition

In a recent study, curcumin was encapsulated into niosomes at different pH values. At lower (pH 3.0–7.0) and higher (pH 9.0) pH values, spherical and irregular niosomes were formed, respectively. The highest entrapment efficiency was observed at pH 3.0 (75.23 ± 2.85%). Thus, an acidic medium is a conducive environment for the incorporation of curcumin into niosomes [53]. There was no notable influence on the vesicle size prior to the storage of the niosomes when the pH was altered during the hydration process. However, evident effects could be observed on the rigidity of the vesicle structure during storage [30]. The pH during the hydration process of the drug delivery vesicle had a significant effect on the stability of the curcumin. In acidic media, curcumin is rigid, with better stability and negligible deterioration [54]. In contrast, a few existing studies show that the stability and rigidity of the curcumin structures are reduced at neutral and alkaline pH levels [55]. Compared to naturally transpiring curcuminoids comprising demethoxy-curcumin, bisdemethoxy-curcumin, and curcumin, pure curcumin is more prone to chemical degradation in an alkaline solution (pH ≥ 7.0). In contrast, it is susceptible to the formation of relatively large crystals ranging from to 10–50 μm in an acidic medium (pH < 7.0), which in turn are subject to sedimentation [56]. This is a consequence of the modification of the molecular structure of curcumin at various pH values. Another factor potentially affecting the drug entrapment efficiency is the pH of the hydration medium. Flurbiprofen, for example, has a higher entrapment rate at an acidic pH (maximum of 94.6% at pH 5.5). Flurbiprofen's entrapment effectiveness increases when the pH lowers from 8.0 to 5.5, although it decreases dramatically at pH 6.8 [57]. The non-target toxicity of chemotherapy is a key drawback limiting the dose and therapeutic window, which niosomal formulations sensitive to pH values can help to overcome. In a study aimed at the efficient treatment of breast cancer, tamoxifen citrate-incorporated niosomes were integrated into CH/glyceryl monooleate to form an in situ pH-responsive hydrogen delivery system with the optimum formula consisting of 0.67% (w/v) of CH and 0.27% (w/v) of glyceryl monooleate. A spectacular regulated release of tamoxifen citrate was observed in Ehrlich cancer mice as a model with an entrapment efficiency and drug release of 88.9% and 49.2%, respectively [58]. In a similar study, a pH-sensitive niosomal formulation of mitoxantrone using cholesterol hemisuccinate instead of cholesterol and a PEG-poly (monomethyl itaconate)-CholC6 copolymer to achieve pH sensitivity and stability was investigated for a human ovarian cancer cell line, umbilical vein endothelial cells, and a breast cancer cell line. These synthesized niosomes exhibited good stability at neutral pH values. However, in the acidic pH of tumor tissues, they expelled their therapeutic moiety content [59].

### 7.3 Nature of surfactant

The primary advantage of traditional non-ionic surfactants is their straightforward chemical modification, as this can be utilized to boost the selectivity of drug release towards specific organs without any harm to the surrounding healthy cells. By altering the structural ingredients of this nanostructure, the characteristics and efficacy can be tuned to achieve the desired membrane flexibility and surface charge [42]. When a charge is present, the inter-lamellar distance between consecutive bilayers in a multilamellar vesicle structure increase, resulting in a larger total entrapped volume [14]. The hydrophilic-lipophilic balance (HLB) was developed to determine which emulsifier would operate best in the oil phase of emulsified product. In terms of product quality and production, calculating the HLB value of a surfactant is critical, as it is widely recognized for having a significant impact on the formation and characteristics of vesicles. Theoretically, HLB values can be estimated or determined empirically. The experimental approach is lengthy and arduous, and was first described in 1949 by William Griffin [60]. Specifically, the HLB levels were calculated using formulas based on analytical or compositional data. The formula used to obtain the approximate values for most polyhydric alcohol fatty acid esters is shown in Equation (1) as follows:

(1) HLB = 20 (1- S/A)

Where *S* is the ester saponification number and *A* is the acid number [61].

Owing to the decrease in surface free energy with an increase in surfactant hydrophobicity, an increase in the HLB value of surfactants leads to an increase in the mean sizes of the niosomes. The niosome bilayers can exist in either liquid or gel conditions. The temperature, surfactant type, and cholesterol play important roles. In the gel state, the alkyl chains are neatly organized, but in the liquid state, they are disordered. The gel and liquid phase transition temperatures of the surfactant influence the entrapment effectiveness. Surfactants with an HLB value of 14–17 are not acceptable for niosomal preparations [14], whereas HLB values ranging from 4 to 8 can produce vesicles [62]. To minimize the influences of the variable hydrophilic head groups of the non-ionic surfactants on vesicle characteristics, all surfactants may be chosen from the Span family (with the same hydrophilic head group but a distinct lipophilic chain). The cholesterol level is also a determinant of the response surface design of niosomes. Owing to interactions with the non-ionic surfactants, the quantity of cholesterol in a vesicle can affect the vesicle's behavior and shape [63]. In one study, this was proven by formulating a niosome with a ratio of 3:2 of Tween 80: Span 80, with an HLB value of 10.72. The disintegration of this niosomal formulation after sonication indicated the amount of added cholesterol, which was highly dependent on the HLB value of the synthesis. In general, as the cholesterol concentration in the suspension elevates, the niosome becomes stiffer, and can withstand sonication as a consequence [13].

### 7.4 Temperature of dispersion medium

The temperature of the dispersion medium during the hydration process has an impact on the sizes and forms of niosomes. The temperature of the hydration solution should be higher than the gel-liquid phase transition temperature. The assembly of surfactants into vesicles and modulation of the vesicle shape are both affected by temperature changes. The adjustment is generally accounted for based on the hydration period and amount of the hydration medium. Fragile niosomes and drug leakage difficulties may occur if the hydration temperature, duration, and hydration medium volume are not correctly chosen [14]. A study on Tween-curcumin niosomes showed that the obtained niosomes were rigid at room temperature but slowly degraded to irrevocable micelles at elevated temperatures [64]. In addition, the fluorescence intensity of the curcumin decreased as the formation temperature increased. This may have been caused by a loss of energy through non-radiative processes. This suggests that the curcumin within a vesicle detects a stiffer and more viscous environment as the temperature increases, whereas the curcumin in the micelles does not. The progressive shift of the curcumin placement in the vesicle from a polar to a partly non-polar environment might explain the high anisotropy of curcumin with increasing temperature for formulations generating vesicles. Another intriguing finding is that the anisotropy values for various formulations differ significantly at low temperatures; however, at high temperatures, the anisotropy values are approximately equal. The curcumin in vesicles has anisotropy values similar to those of the curcumin in micelles at high temperatures [64].

### 7.5 Coating of niosome

A study was conducted on bare and CH-coated niosomes. These niosomes were formed using a modified version of a heating method in which three factors (Span 60, Tween 60, and cholesterol) were altered into seven different formulations. The purpose of the CH was to adjust the surface of the curcumin niosome. The CH vesicles were smaller in size. Thus, the CH-coated niosome established a sustained curcumin release profile. These results confirmed that coating curcumin niosomes with CH effectively improves the blood-brain barrier permeability of curcumin relative to free curcumin and bare curcumin niosomes. This was supported by the fact that CH is a positively charged element. Hence, CH can react with negatively charged elements in a biological environment, resulting in enhanced permeability [65]. In another study, hydrophobin was utilized as a coating for niosome vesicles. This study strongly demonstrated the merits of hydrophobin-coated niosomes relative to PEG-coated niosomes. The hydrophobin-coated niosomes were prepared using a thin film hydration method in which Span 40, Tween 40, and cholesterol were used in various formulations. Compared to the marketed anti-cancer medications, hydrophobin-coated niosomes have reduced *in vitro* cytotoxicity. Furthermore, compared to control cells, the hydrophobin-coated formulation displays higher cytotoxicity against cancer cells, namely, the A549, MDA-MB-231, C6, and PC1 cancer cell lines. These proteins may provide protection against immune system detection *in vivo*, thereby preventing an immunological response. Physically coating niosomes with proteins with relevant functionalities is a cost-effective, adaptable, and time-saving technique for creating drug delivery vehicles [39]. Compared to PEG-coated niosomes, hydrophobin-coated niosomes have a better size distribution, greater entrapment efficiency, a more prolonged release profile, superior biocompatibility, and improved anti-cancer effects. Surprisingly, one study showed that the viability percentage of the control cell line was greater than that of several cancer cell lines after treatment with the formulations, indicating that the formulation had a stronger selectivity against cancer cells. As a result, there are promising prospects for creating and synthesizing covered niosomes at a reasonable cost [66].

### 7.6 Functionalized niosomes

The design of a functionalized niosome usually incorporates ligands, antibodies, receptors, cell-penetrating peptides, proteins, sugars, and specialized targeting properties for cancer treatments often situated in the brain, colon, liver, lungs, and breasts. These functionalized vesicles can penetrate the blood barrier. Therefore, they represent a potential remedy targeting cancer cells. In one study, a folic acid-functionalized niosome encapsulated in curcumin and letrozole was synthesized to fight breast cancer. In this study, the lipid-to-drug ratio was manipulated. The lipid-to-drug ratio (10:1) achieved a vesicle with the smallest size but the highest entrapment of both drugs. In addition, this formulation also displayed great stability during storage for a month [67]. In another study, folate-PEGylated niosomes encapsulated in letrozole and ascorbic acid were synthesized to combat breast cancer. In a cytotoxicity study, the vesicle demonstrated high biocompatibility with healthy cells and simultaneously inhibited the metastasis and formation of breast cancer cells (MDA-MB-231 and SKBR3). Flow cytometry studies proved that the folate-PEGylated niosome could accelerate apoptosis in breast cancer cells owing to the synergistic delivery of the dual drug [68]. A new era of research is needed to determine the best chemotherapy dosage in the target area for aggressive brain tumor glioblastomas owing to the low permeability of the blood-brain barrier, as this creates a challenge for medication transportation. According to recent *in vivo* experiments, the functionalized vesicles can specifically target brain tumor cells owing to their capacity to cross the blood-brain barrier. The creation of this potent vesicle was particularly appealing for targeting the glioblastoma cells of brain tumors via niosomal surface modifications with chlorotoxin, a peptide obtained from the venom of the Israeli yellow scorpion (*Leiurus quinqustriatus*), as well as the alkylation drug temozolomide. These modifications permit the niosomal formulation to undergo active targeting exhibiting a 3.04-fold increase in the temozolomide accumulation in the cerebri. The chlorotoxin-functionalized niosomes with temozolomide encapsulation achieve an entrapment efficiency of 79.09% with a vesicle size of 220 nm. As a consequence of surface modification, improved penetration of the therapeutic moiety into the brain to fight tumor cells is expected [69].

## 8 Cytotoxicity of curcumin niosome

Cytotoxicity is the toxicity brought on by a chemotherapeutic agent's interaction with live cells. Drug delivery systems like niosomes that encapsulate therapeutic moiety are known to have harmful effects on the body's organs, and small particles have a greater ability to injure cells by entering them. Additionally, niosomes' surface charges can make them more reactive with cells and proteins. To assess cellular metabolic activity as a sign of cell viability, proliferation, and cytotoxicity, the 3-(4,5-dimethylthiazol-2-yl)- 2,5-diphenyl-2H-tetrazolium bromide (MTT) assay is employed. This colorimetric test relies on the transformation of purple formazan crystals into a yellow tetrazolium salt [70]. In context of the cytotoxicity studies, the cytotoxicity of curcumin niosome is analysed for human ovarian cancer A2780 cell line using MTT assay post 24 h incubation. The results exhibited that curcumin niosome possess elevated cell killing rates in relative to free curcumin. With more than 90% of cells were still alive after being incubated with free curcumin for 24 h while only around 50% of cells survived treatment with 12.5 μm curcumin niosomes. When curcumin was employed at a greater dosage (25 μm), the cytotoxicity of curcumin niosomes was more powerful that it killed more than 80% of the cells whereas free curcumin only killed 20% of cells. Curcumin niosomes demonstrated exceptional cytotoxicity when compared to free curcumin that is a consequence of greater aqueous curcumin solubility and increased cellular absorption in niosomes [7]. To test its cytotoxicity on HFF normal fibroblast cell line, several concentrations (ranging from 0.5 to 15 g/ml) of free curcumin, curcumin niosome and blank niosomes were analysed. In the presence of blanks niosomes, free curcumin and curcumin niosomes, the viability of the cells did not significantly decline over the course of 24 h due to surface charge presented by the curcumin niosome is −0.73, which was very close to zero [28]. The cytotoxicity of curcumin loaded niosomes with calcium alginate shell (Al-NioC), curcumin niosome (NioC), free curcumin and blank niosomes were assayed. As a result, the Al-NioC formulation and NioC demonstrated considerable toxicity but stronger impact was noticed for Al-NioC. Al-NioC may enhance the binding of nanocarriers to cancer cells and boost the release rate in these cells together with alginate on the surface of niosome vesicles [71]. The effect of Al-NioC and NioC formulations was tested on two cell lines SKBR3 and MDA-MB-231 where the repercussions on the SKBR3 cell line was relatively potent. Al-NioC and NioC are less hazardous to the MCF10A cell line than curcumin, as evidenced by the toxicity levels of Al-NioC and NioC. Additionally, as the concentration of the aforementioned samples was increased, cell viability dropped, demonstrating notable variations in toxicity among all samples [72].

## 9 Non-clinical and clinical application of niosome in cancer treatment

### 9.1 Non-clinical application

To commercialize or launch a specific pharmaceutical drug, it is vital to satisfy both non-clinical and clinical trial standards. Clinical tests carry massive weight in analyzing the human responses to any therapeutic moiety. Nevertheless, non-clinical tests are necessary before investigating and studying the effectiveness of drugs and their safety in humans. Numerous niosomal formulations have been studied to boost efficiency minimize toxicity, most notably for cancer. Table 3 outlines the *in vivo* model from this perspective. From this table, we can conclude that niosomes are a viable carrier system for hydrophilic, hydrophobic, and lipophilic medicines for therapeutic purposes. Based on investigations conducted on *in vivo* models, niosomes possess a large drug payload capacity, regulated therapeutic moiety release, and higher entrapment. Additionally, *in vitro* tests of these published articles mostly demonstrate that the niosomal delivery system is able to sustain drug release more successfully than a free drug solution. This extends the duration of the drug release profile at the target site. Furthermore, it has been shown that a long-circulating colloidal delivery system with increased bioavailability has been present in the animals employed for testing, including rats and mice. This may be because the concentration of the drugs utilized could be controlled and maintained above the minimal effective concentration for 24–48 h.

**Table 3:** Evaluation of *in vivo* testing of various niosome formulations for cancer treatment in animal models that encompasses the ingredients of the formulation, synthesis method, type of drug encapsulated, *in vivo* model employed, animal species used for testing, indication of human cell line and the characteristics of the vesicles, including entrapment efficiency, average diameter, and *in vitro* release studies.

Ingredients of Formulation	Synthesis Method	Drug	*In vivo* Model	Animal used	Indication	Characteristics of Vesicle			References
						Average Diameter (nm)	Entrapment Efficiency (%)	*In vitro* Release Results	
Span 60, cholesterol, solulan, sodium dicetylphosphate	–	Doxorubicin	Mouse adenocarcinoma model	Male NMRI mice	Ovarian cancer	154	40%	aAt pH 7.0, 28.92% (4 h) 37.10% (8 h) 45.68% (24 h)	[73]
(PEG)-ylated paramagnetic niosome bearing *N*-palmitoyl glucosamine	Sonication	Gadomenate dimeglumine	Human carcinoma xenograft model	Male nude mice (nu/nu)	Prostate carcinoma	186	4.60%	aAt tumor, 37.68% (6 h) 47.43% (12 h) 52.68% (24 h) At liver, ∼21.38% (6 h) 40.68% (12 h) 25.50% (24 h) At muscle, 12.38% (6 h) 27.93% (12 h) 22.87% (24 h) At brain, 9.17% (6 h) 18.18% (12 h) 24.56% (24 h)	[74]
Span 80, cholesterol, and diacetyl phosphate	REV	Daunorubicin hydrochloride	Dalton's ascitic lymphoma-bearing mouse cells	Male Swiss albino mice	–	700–1000	69%	–	[75]
Spans 40, 60, 80, Tween 20, 40, 60 and cholesterol	Thin film hydration	Β-carotene	RAT-1immortalized fibroblasts	–	Human adeno-carcinoma	338.4–440.3	9.7%–51.6%	–	[76]
PEGylated niosomes modified with transferrin (Tf)	Thin film hydration	Hydroxy camptothecin	S180 cells in mice	Male Wistar rats	Erythroleukemia	116	Tf-PEG-NS 93% PEG-NS 93.6% *n*-NS 94.1%	aAt pH 7.4, Tf-PEG-NS 65.09% (12 h) 79.45% (24 h) 87.72% (36 h) 90% (48 h) 97.5% (60 h) PEG-NS, 59.63% (12 h) 74.63% (24 h) 83.91% (36 h) 89.36% (48 h) 93.46% (60 h) *n*-NS 37.00% (12 h) 52.00% (24 h) 55.55% (36 h) 62.09% (48 h) 65.36 (60 h)	[77]
Hydrochloric acid, Tween 80, Span 20	Emulsion-evaporation	Monostearin	Mouse breast tumorcell (4T1)	Male Kunming mouse	Breast cancer	38.2–47.2	56.5%–94.3%	–	[52]
Span 40/Span 60/Span 80/Tween 20 and cholesterol	Thin film hydration	Tamoxifen citrate	Ehrlich carcinoma mice model	Swiss albino mice	Breast cancer	212.2	37.4%–92.3%	aAt pH 7.4 F2 88.48% (50 h) F3 71.20% (50 h) 85.82% (100 h) 98.40 (150 h) F5 67.04% (50 h) 72.48% (100 h) 72.80 (150 h) F7 63.7% (50 h) 94.45% (100 h) 100.00% (150 h) F8 50.62% (50 h) 73.10% (100 h) 91.19% (150 h) F10 42.24% (50 h) 44.76% (100 h) 48.39% (150 h)	[78]
Span 60, Tween 60, cholesterol-PEG 600 and diacetyl phosphate	Thin film hydration	Vinblastine	C57BL/6 mice model	Winstar rats	Lung cancer	234.3	99.90%	At 37 °C in 95% CO, Free VB, 5.3% (48 h) 7.4% (72 h) PEG-niosomal VB, 10.4 (48 h) 13.3 (72 h)	[79]
Span 60, cholesterol and diacetyl phosphate	Reverse phase evaporation	Withaferin	Higuchi and Korsmeyer Peppas model	Swiss Webster female albino mice	Human cervical cancer	278	87.30%	At pH 7.4, 85% (12 h)	[80]
Cholesterol and Span 60	Thin film hydration	Tamoxifen	Ehrlich carcinoma mice model	Swiss albino female mice	Breast cancer	325.5	91.20%	At pH 7.4, 45.4% (8 h)	[81]
Span 60, Tween 60 and cholesterol	Thin film hydration	Melittin	LM-3 xenograft tumor model	BALB/C inbred mice	Breast cancer	121.4	79.30%	aAt pH 5.4, 51.3% (12 h) 72.2% (72 h) At pH 6.5,44.9% (12 h) 80.8% (72 h) At pH 7.4, 43.5% (12 h) 92.1% (72 h)	[82]

a *The data is directly extracted from articles using WebPlotDigitizer* [83].

### 9.2 Clinical application

Many studies on niosomal formulations encapsulating various anti-cancer drugs have been published in recent years. Many authors have investigated different niosomal formulations to improve the effectiveness and limit the toxicity of the vesicles. For instance, Span 80 vesicles with a 63% loading efficiency of drug were shown to successfully transfer drug into the cytoplasm of the tumor cells with direct contact of cellular membrane and vesicles membranes [84]. Table 4 lists the anti-cancer niosomal formulations, drugs, cell lines, and their characteristics. As summarized in Table 4, most niosomal formulations consist of non-ionic surfactants and cholesterol. Cholesterol is a key component in niosomes [22]. Most formulations contain it in a 1:1 M ratio to avoid vesicle aggregation, i.e., by including molecules to stabilize the system against the development of aggregates owing to repulsive steric or electrostatic forces [85]. In many of these clinical experiments, the niosome formulation produce vesicles with a nanometric size ranging from 100 to 500 nm and a spherical morphology. Moreover, the entrapment efficiency of the drug delivery system significantly increases after the therapeutic drug is incorporated into the niosome relative to the free drug solution. Additionally, the clinical application of niosomes shows great efficacy in human cancers generated from tumors located in the breast, ovary, lungs, and colon. The *in vitro* release of therapeutic drugs with anti-cancer properties is highly pH-dependent. Overall, these findings offer proof-of-concept for the therapeutic benefit of encapsulating anti-cancer drugs in niosomal formulations.

**Table 4:** Evaluation of niosomal formulations encapsulating anti-cancer drugs for the treatment of multiple cancer types including details on the formulation ingredients, synthesis method employed, specific drug encapsulated, cell line used for testing, indication of human cell, and the characteristics of the vesicles, including entrapment efficiency, particle size, and zeta potential.

Ingredients	Synthesis Method	Drug	Cell line	Indication	Characteristics				References
					Particle Size (nm)	Zeta Potential (mV)	Entrapment Efficiency (%)	Release Results	
Pluronic L64, Tween 60 and cholesterol	Thin film hydration	Doxorubicin	K562	Leukemia	147–379	–	87.86–98.31	At pH 7.4, T60-D, (26%) L64-D, (39%)	[86]
Tween 60, Span 60, Span 80, cholesterol,d iacetyl phosphate	Thin film hydration	Morin hydrate			T60: 181.3 S60: 131.3 S80: 189.1	T60: −7.40 S60: −19.05 S80: −16.6	T60: 93.4% S60: 79.7% S80: 71.2%	aAt pH 7.4 T60: 798.72 μm (12 h) 908.87 μm (24 h) 966.29 μm (48 h) 1049.43 μm (72 h) S60: 721.58 μm (12 h) 797.44 μm (24 h) 850.57 μm (48 h) 890.86 μm (72 h) S80: 584.4 μm (12 h) 634.58 μm (24 h) 666.29 μm (48 h) 693.71 μm (72 h) At plasma, T60: 642 μm (12 h) 715.82 μm (24 h) 772.55 μm (48 h) 829.28 μm (72 h) S60: 387.78 μm (12 h) 415.18 μm (24 h) 440.72 μm (48 h) ∼474.72 μm (72 h) S80: 214.72 μm (12 h) 234.00 μm (24 h) 245.52 μm (48 h) 270.18 μm (72 h)	[87]
PEG-Poly (monomethyl itaconate)-CholC6 (PEG-PMMI-CholC6) and cholesterol hemisuccinate	Thin film hydration		MCF-7, OVCAR-3 and HUVEC	Breast and ovarian cancer	156.6–186.3	(-12.1) – (−20.6)	70.9%–73.2%	At pH 6.5, PEG-PMMI-CholC6 : 65.4% CHEMS: 70.2% At pH 7.4, PEG-PMMI-CholC6 : 20% CHEMS:10%	[59]
Span 60, cholesterol and diacetyl phosphate	Thin film hydration	Imatinib Mesylate	MCF-7, HCT-116 and HePG-2	Colon adenocarcinoma	425.36	−62.4	82.96%	aAt pH 7.4, initial:18.93% cumulative: 89.45%	[88]
Span 60 and cholesterol	Thin film hydration	Cisplatin	BT-20	Breast cancer	489.3	23.4	41.2%	aAt pH 7.4, 32% (1 h) 24% (48 h)	[89]
PEG2000, Span 20 and cholesterol	REV	Silibinin	T47D	Breast cancer	380	–	70.61%	At pH 7.4, With tamoxifen: 17.797% Niosome: 22.034	[90]
Span 80 and cholesterol	Thin film hydration	Tamoxifen-curcumin	MCF-7	Breast cancer	159.45		TMX: 98.37% CUR: 96.40%	–	[91]

a The data is directly extracted from articles using WebPlotDigitizer [83].

### 9.3 Targeted cancer treatment receptors

**Fig. 3:**
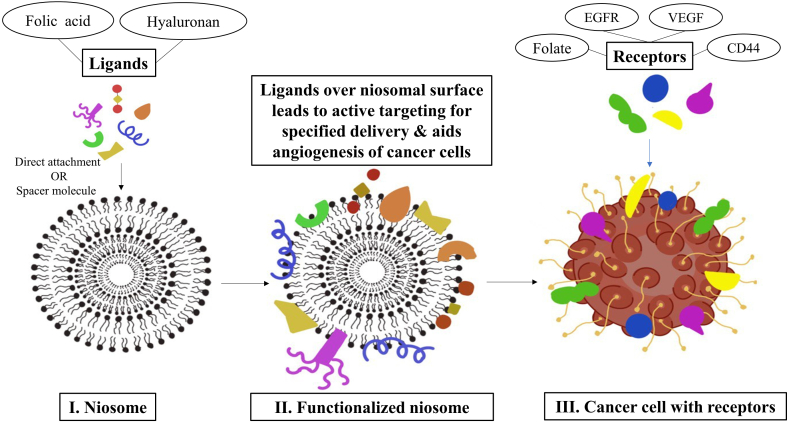
Functionalization of bare curcumin niosomes with ligands for active targeting of cancer cells as niosomes encapsulating curcumin are modified by attaching specific ligands onto their surface to specifically bind with receptors on the surface of cancer cells to facilitate enhanced drug delivery.

Non-directed and non-specific cancer treatments may induce substantial adverse effects and systemic toxicities. In such cases, providing precise and targeted delivery of the therapeutic compounds is essential. Targeting biomarkers that are overexpressed in tumor cells permits tailored delivery of the cytotoxic payload to malignant tissues, decreasing side effects and enhancing the therapeutic index [92]. Fig. 3 shows the receptors and their ligands as expressed in cancer cells. Several ligands for various receptors bind and attach to immune cells and may slow tumor development. When these ligands are bound onto the surface of the niosome, they aid in the orientation of vesicle formation, and permit more accurate targeting and efficient drug delivery. Hence, the prime receptors for active and precise targeting include the cluster of differentiation 44 (CD44), folate receptor, vascular endothelial growth receptor (VEGF), and epidermal growth factor receptor (EGFR).

CD44 is a multi-functional structural protein that acts as a cell surface adhesion receptor and aids in the proliferation, migration, and angiogenesis of cells [93]. With hyaluronic acid's ability to precisely bind with CD44, their potential for enhanced permeability and retention allows hyaluronic acid-conjugated niosomes to actively target tumors and improves medication absorption by cancer cells through the hyaluronic acid-CD44 receptor-mediated endocytosis pathway [94]. A study in which curcumin niosomes were coated with hyaluronic acid showed that protein adsorption and the rate of macrophage uptake was reduced in contrast to the non-specific cell uptake mechanism of uncoated niosomes [95]. The hyaluronic acid coating alleviated the negative charge causing decreased protein adsorption, and facilitated targeted delivery to cancer cells with CD44 receptors [96].

Folate receptors are naturally existing surface proteins that bind to folate or folate conjugates with a strong affinity [97]. This receptor is overexpressed on the surface of human cervical cancer cells, as claimed in medical research, resulting in extensive research on this receptor. PEG is the most common polymer used for folic acid [98]. Owing to their active targeting of tumor tissues via folate receptor-mediated endocytosis, curcumin-encapsulated folic acid niosomes have significant cellular absorption and tumor-killing impacts, as the nucleus of cancer cells is destroyed and the potential of mitochondria in cells abruptly falls as the cancer cells undergo apoptosis [99].

EGFR is a transmembrane protein receptor that is overexpressed in various cancer cells. EFGR is normally tagged HER1 and ErB-1 [100]. These receptors are also expressed in 85% of nasopharyngeal cancer patients. Monoclonal antibodies, such as cetuximab and nimotuzumab, target the EGFR protein expressed on the malignant cells. Taking these into account, a doxorubicin-encapsulated niosome can capture the EFGR-targeted cells that penetrate the drug delivery system. The vesicle is synthesized by a thin film hydration method using the surfactants pluronic P123 and cholesterol, resulting in an entrapment efficiency and vesicle diameter of 88% and 200 nm, respectively. In one study, the drug release profile of this nanoparticle was sustained for 24 h at pH 7.4 [101]. In a similar study, niosomal curcumin + microRNA-34a (miR-34a) acted as a successful treatment for cancer cells after testing in 4T1 xenografted Balb and C mouse tumor models [102]. This was supported by a study on matrix metalloproteinase-7 dependent gastric cancer cells, where a notable decrease in miR-34a and increase in EGFR relative to normal tissue were observed [103].

VEGF is a signaling protein that allows for the production of vessels that carry blood, such as in angiogenesis and vasculogenesis [104]. The formation, angiogenesis, and progression of cancers, particularly liver and breast cancers, are significantly influenced by VEGF [105]. The synergistic effects of paclitaxel (a chemotherapy medication) and VEGF small interfering RNA can decrease cancer growth. By effectively inhibiting VEGF expression and inducing cancer cell apoptosis, this combination produces considerable antitumor activity both *in vitro* and *in vivo* [106]. These receptors are clearly distinguished in Table 5.

**Table 5:** Cataloging of target receptors and their corresponding ligands expressed on cancer cells.

Receptor	Ligands	Cells	References
Cluster of differentiation 44 (CD44)	Hyaluronan (most common)	Glioblastoma multiforme cell	[93]
		4T1 cells mimic human breast cancer	[52]
	Osteopontin	Leukemia cancer stem cells	[107]
	Ezrin		[108]
D44 variant isoforms (CD44v)	Selectin	Colon carcinoma cells	[109]
Folate	Folic acid	Epithelial ovarian cancer	[110]
		Carcinoma head and neck	[111]
		Ovarian and breast cancer	[97]
		Colon cancer	[112]
Epidermal growth factor receptor (EGFR)	EGF, TGF-α	Lung cancer	[113]
	EGF, TGF-α	Breast, colorectal and lung cancer	[100]
Vascular endothelial growth receptor (VEGF)	Tivozanib	Kidney cancer	[104]
	Bevacizumab	Colorectal cancer	[114]

### 9.4 Cancer chemotherapy

Various therapeutic moiety delivery technologies for cancer chemotherapy have been investigated and analysed. There are multiple difficulties in cancer treatment, such as non-targeted delivery, short drug release profiles, and drug resistance tumor interactions [18]. Hence, cancer treatment techniques vary to support the efficiency and effectiveness of treatments targeting cancer cells. Table 6 lists these techniques. Owing to the specific characteristics of the tumor milieu not typically present in healthy tissues, passive targeting makes it easier for niosomes to be deposited into the tumor microenvironment [22]. Furthermore, active targeting facilitates the uptake of the niosomes by tumor cells. Adaptable molecules can be used to create functionalized drug vesicles for targeting tumor cells [115]. Stimuli-responsive targeting is vital to developing future niosomal formulation medication technologies, as they are sensitive to signals oriented from physiological systems or abnormalities originating from pathological defects that could potentially interact with the biological environment [102]. Dual targeting is a novel idea in which the niosomal formulation synthesizes a dual receptor-targeted formulation to improve the specificity of cellular targeting without destroying healthy adjacent tissue cells [116]. Moreover, dual targeting with a dual drug-loading strategy acts as a combination therapy using two or more medications and encourages synergism, and the vehiculation into multi-functional niosomes has demonstrated a great deal of potential for cancer treatment [117]. Another innovative approach to fighting cancer is immunotherapy, which uses the immune system to combat the disease. To enhance the effectiveness of immunotherapy and reduce adverse effects, immune-simulating drug-encapsulated niosomes should be properly supplied to the target region [118].

**Table 6:** Treatment techniques for efficient drug delivery in cancer treatment focusing the principles underlying each treatment technique, along with their respective advantages.

Treatment Techniques	Principle	Advantages	References
Passive Targeting	Permits nanoparticle localization in tumor microenvironment	**•**Promotes tumor nanovector deposition. •Increased retention of permeability	[119]
Active Targeting	Delivers therapeutic agent focusing on specific sites	**•**Improves delivery rate. •Reduces side effect •Enhanced accumulation and retention	[120,120]
Stimuli- Responsive Targeting	Diagnostic agent releases from drug delivery systems receptive responsive of apt stimuli	•Cheaper option for stimuli sensitive treatment cancer	[121]
Dual Targeting	Formulation with dual receptor to specify cell targeting	**•**Improved and better antitumoral activity **•**Enhanced specificity and focus of drug	[122]
Dual Targeting with Dual Drug Loading Strategy	Drug delivery systems binds two different ligands and drugs to specify cell targeting	**•**Improved specificity and synergistic activity to fight cancer. •Reduces toxicity	[40,117]
Immunotherapy	Regulates human immune system to fight against cancer cells	**•**Develop vaccine to activate tumor bonded antigens •Promotes drug load delivery •Elevates efficacy of antitumoral activity	[123]

## 10 Application of curcumin niosome in cancer treatment

### 10.1 Ovarian cancer

Ovarian cancer is a general gynecological cancer that contributes to mortality. Despite this, an effective therapy for this disease remains elusive owing to several obstacles, including the fact that prior discovery is almost impossible as the symptoms in the early stage are minimal. In addition, ovarian cancer has a high chance of relapse and is noticeably resistant to chemotherapy treatments [124]. Current cancer therapy research is focused on developing curative healing agents to target ovarian cancer. In an investigation of the A2780 cancer cell line, free curcumin showed poor pro-apoptotic and cytotoxic effects compared to curcumin niosomes. This demonstrated that the curcumin encapsulated in niosomes exhibits better cellular absorption and bioactive protection. The intracellular limits of the curcumin niosome increased 1.43 times after incubating for 4 h relative to free curcumin. After 24 h of treatment with curcumin, a cell cycle method was used to analyze the cancer inhibition mechanism of the curcumin-incorporated niosomes. The apoptotic activity of the ovarian cancer cells escalated, proving that the curcumin niosome represents a great alternative treatment. Moreover, a substantial cell cycle arrest was noticed in the S phase. These findings not only prove the feasibility of niosomes in facilitating the delivery of curcumin to treat cancer; they also show that niosomes can simultaneously be appropriately operationalized to accurately locate the site of the tumor with a higher rate of sensitivity. In addition, the curcumin niosome prevents the tumor from spreading extensively, e.g., all over the body [125].

### 10.2 Breast cancer

Breast cancer is a major malignancy in humans, and women are more likely to develop it [126]. The rate of fatality caused by this cancer is increasing worldwide. Minimally, 1.3 million women worldwide are confirmed to have cancer in or around the breast every single year [67]. Nanomedicine focused mainly on chemotherapy is considered the main treatment for breast cancer, especially post-surgery [127,128]. Similar to other treatments, chemotherapy has side effects after treatment. However, the level of chemical resistance present in chemotherapy treatments poses the risk of impeding and obstructing the healing process. The pattern regarding the severity of the chemically resistant behavior can be observed in the initial stage of chemotherapy, and sometimes following an assuring initial response to treatment [129]. In a related study, niosome calcium alginate was employed as a drug delivery system for curcumin and was tested on MDA-MB231 and SKBR3 breast cancer cell lines. The gene expression levels of these carriers were assessed to determine the efficacy of this unique strategy in treating breast cancer. The curcumin encapsulated in the niosome calcium alginate promoted cancer cell mortality and apoptotic effects in MDA-MB231 and SKBR3 cells [130].

### 10.3 Liver cancer

Liver cancer is one of the most common cancers globally [131]. Most patients with liver cancer are diagnosed at a convoluted stage. Thus, non-surgical cancer treatments, such as systemic chemotherapy, are in massive demand [132]. Single-agent medicines, such as DOX, cisplatin, sorafenib, and 5-fluoroucil, are highly rated regarding the effectiveness in beating liver cancer. However, the response rate of a highly efficient single-agent medicine is just 10%, with no discernible benefit to the overall survival of humans. This may be attributed to the noxious level and low chemoresistance response [133].

**Fig. 4:**
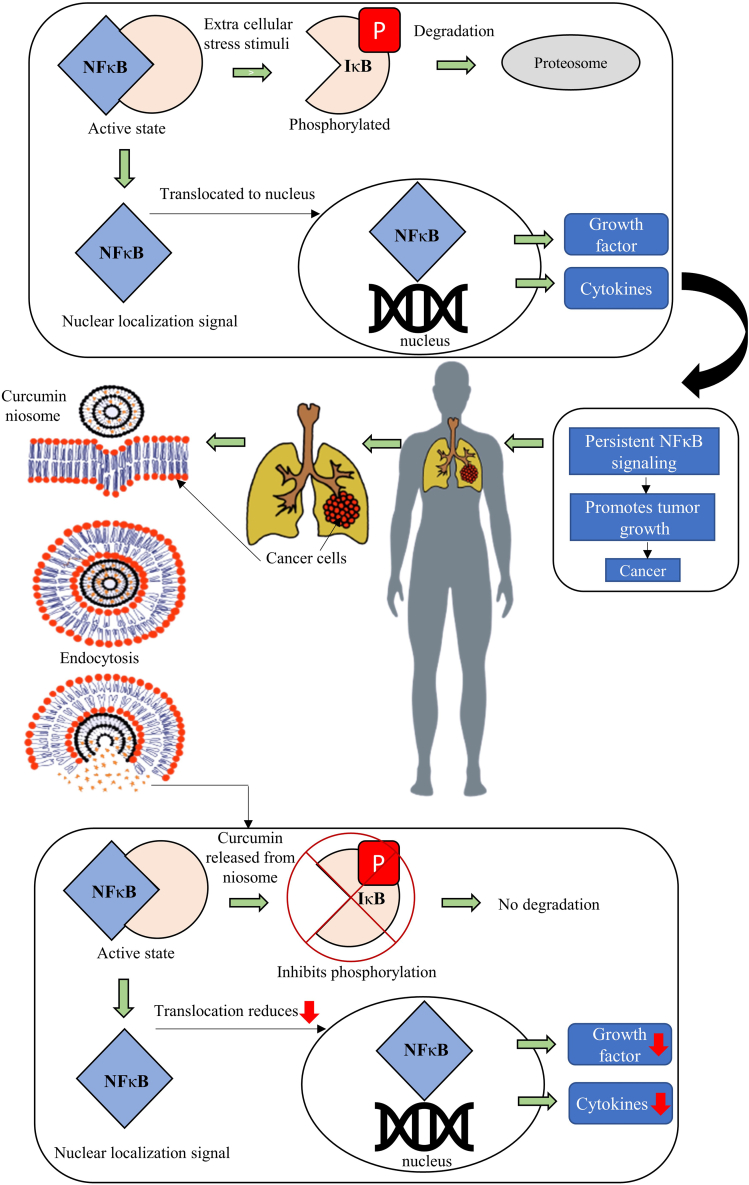
Illustration of molecular mechanism of curcumin targeting the NFκB signaling pathway inducing apoptosis and inhibition of cancer cell metastasis that encompasses the activation of NFκB and subsequent downstream signaling events leading to cell survival, inflammation, and metastasis.

An interesting study was performed by incorporating DOX and curcumin as a promising strategy for curing liver cancer [134]. Vesicles carrying the drug were prepared using a microfluidization technique under high pressure. A cell cytotoxicity assay revealed that the generated DOX/Curcumin nanoparticles were non-toxic. The DOX/Curcumin-nanoparticles represent vesicles incorporating dual drugs together, whereas DOX-nanoparticles and curcumin-nanoparticles represent nanoparticles only encapsulate single drugs (i.e., either DOX or curcumin). When comparing DOX/Curcumin nanoparticles to DOX-Curcumin nanoparticles, the *in vitro* release experiments demonstrated a prolonged drug release pattern. The DOX/Curcumin nanoparticles had synergistic effects compared to a single DOX-Curcumin nanoparticle, as evidenced by increased cytotoxicity and apoptotic activity in HepG2 cells, reduced cytotoxicity in L02 cells, and enhanced tumor growth inhibition *in vivo*. These findings suggest that delivering DOX and curcumin simultaneously via DOX/Curcumin nanoparticles is a promising treatment for liver cancer [134]. Table 7 focuses on the characteristics of curcumin niosomes in cancer studies, whereas Fig. 4 provides simplified representation of curcumin's molecular mechanism targeting the Nuclear Factor Kappa B (NFκB) signaling pathway. It aims to convey the general concept of curcumin's effects on apoptosis and metastasis inhibition in cancer cells. Curcumin exerts its action on the NFκB signaling pathway, where NFκB acts as a transcription regulator, normally binding with the inhibitor of NFκB (IκB) [135]. In response to extracellular stress stimuli such as environmental toxins, UV rays, and pathogens, the IκB molecule undergoes phosphorylation and subsequent degradation by the proteasome [136,137]. Consequently, the unmasked NFκB molecule reveals a nuclear localization signal, facilitating its translocation to the cell nucleus [138]. Once in the nucleus, NFκB binds to specific DNA sequences, regulating the expression of multiple genes associated with the production of growth factors and cytokines [139]. Persistent NFκB signaling can promote tumor growth, contributing to cancer development. However, curcumin exhibits chemo preventive effects by inhibiting the phosphorylation of IκB in tumor cells. This inhibition reduces the translocation of NFκB to the nucleus [19]. As a result, the diminished activity of NFκB decreases the production of growth factors and cytokines within tumor cells, thereby inhibiting the proliferation of premalignant cells and impeding their progression into cancerous cells [140].

**Table 7:** Comprehensive analysis of curcumin niosome studies on cancer cells summarizing details on the ingredients of the formulation, synthesis method employed, cell line used for testing, as well as the key characteristics of the vesicles, including entrapment efficiency, polydispersity index, *in vitro* release studies and zeta potential.

Composition/Formulation of Niosome	Synthesis Method	Abbreviation of Formulation of Vesicle	Cell Lines	Characteristics of Vesicle				References
				Zeta potential (mV)	PDI	Entrapment Efficiency (%)	Release results	
Polyethylene glycol methyl ether acrylate conjugated with cysteine and curcumin	–	PEG-Cys–NIO–CU	(MCF 7) Human breast cancer cell line	∼+6.5 mV (pH 5.5) ∼-1.5 mV (pH 7.0)	–	–	*At pH 5, 65.13% (1 h) 83.30% (2 h) At pH 7.0, 11.96% (1 h) 15.45% (2 h)	[141]
Tween 80/cholesterol with curcumin and doxorubicin hydrochloride	Thin film hydration	Niosome with CUR + DOX encapsulated	(HeLa) human Cervical carcinoma cell line	−24 ± 2 mV	0.265	CUR = 90% DOX = 25%	*At pH 5.5 (DOX), 70.7% (12 h) 80.13% (24 h) At pH 7.4 (DOX), 68.34% (12 h) 76.37% (24 h) At pH 5.5 (CUR), 16.45% (12 h) 24.45% (24 h) 56.16% (72 h) At pH 7.4 (CUR), 16.45% (12 h) 23.80% (24 h) 51.13% (72 h)	[25]
Span 80, Tween 80 and Poloxamer-188 encapsulated niosome	Solvent evaporation	CUR-NIO	(A2780) Ovarian cancer cells	−25 to −20 mV	–	92.3%	At pH 5.6 (CUR), 48.27% (6 h) 56.89% (12 h) 78.41% (24 h)	[7]
Span 60, cholesterol, DSPE-PEG maleimide with penetrating peptide and polyethylene glycol coated niosome	Thin film hydration	PEGNIO DOX- CUR-tLyp-1	(U87) Human glioblastoma cells	–	0.140–0.175	31.2% (CUR) 22% (DOX) 32.6% (PEGNIO DOX-CUR-tLyp-1)	*At pH 5.6 (CUR), 42.07% (24 h) 61.45% (32 h) At pH 7.4 (CUR), 34.17% (24 h) 35.27% (32 h) At pH 5.6 (DOX), 62.32% (24 h) 74.04% (32 h) At pH 7.4 (DOX), 50.96% (24 h) 68.11% (32 h)	[76]
Cationic PEGylated niosomal formulations containing Tween-60 and cholesterol	Thin film hydration	Dioleyl-3-trimethyla mmonium propane (DOTAP): DSPE-mPEG with paclitaxel (PTX) and curcumin (CUR)	(MCF-7) Human breast cancer cells and (MCF-10 A) Human mammary epithelial cells	+15 mV	–	∼100% for both drugs	*At pH 7.4 (CUR), 25.34.% (12 h) 26.36% (24 h) 29.43% (72 h) At pH 7.4 (PTX), 18.52% (12 h) 19.31% (24 h) 22.82% (72 h)	[49]
Curcumin niosomes shelled with calcium alginate	Thin film hydration	AL-NioC	(SKBR3 and MDA-MB-231) breast cancer cell lines	–	–	80.29%–97.49% (NioCs)	*(FREE CURCUMIN) 73.57% (6 h) 97.14% (24 h) 98.43% (48 h) 98.88% (72 h) (AL-NioC at pH 3) 56.91% (6 h) 72.54% (24 h) 76.19% (48 h) 79.45% (72 h) (AL-NioC at pH 5) 30.32% (6 h) 51.19% (24 h) 59.17% (48 h) 62.78% (72 h) (AL-NioC at pH 7.4) 12.86% (6 h) 30.47% (24 h) 40.07% (48 h) 44.92% (72 h)	[130]

## 11 Research focus for niosomes in the future

**Fig. 5:**
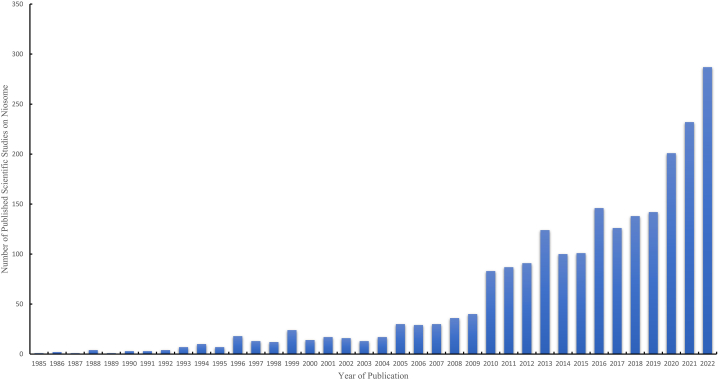
The rising trend of research activity and publication output related to niosomes from 1985 to 2022 in the Scopus database (searched keyword: “niosome,” accessed on June 28, 2023).

Niosomes are undoubtedly a promising and cutting-edge drug delivery technology, and their near future remains highly promising for a variety of therapeutic treatments. As niosomal drug delivery technology remains in its infancy, there are only 2327 publishes scientific studies on niosomes as revealed by the Scopus database (accessed on June 28, 2023). In view of the potential of niosomes, researchers worldwide are exploring niosomes as alternative drug delivery systems. This can be observed in the increase in the number of research articles and studies published on niosomes from 1985 to 2022 (Fig. 5). The highest number of publications, with a total of 287 scientific studies, was achieved in 2022. Most published reports highlight the value of such innovation and aim to synthesize niosomes particularly tailored to meet a range of therapeutic needs. Therefore, greater efforts must be made to direct future discoveries into diversified clinical fields [142]. The formulation, selection of the surfactant, pH condition, temperature, and coating of niosomes are important aspects to consider while designing non-ionic vesicular systems. It is widely recognized that the size, stability, entrapment efficiency, and targeting characteristics of niosomes are directly influenced by these factors. This provides an opportunity to create custom niosomes for desired therapeutic responses. To modify and optimize niosomes for various therapeutic objectives and potential translations, research is ongoing, including experiments with new synthetic materials and therapeutic moieties. The discovery of therapeutic targets for numerous types of cancer has rapidly grown over the past few years, as the target molecules on the niosomes permit greater specificity and selectivity for breast, colon, lung, liver, and many other cancers. In one study, functionalized niosomes were shown to specifically target tumor cells to combat cancer, owing to their capacity to cross the blood barrier and localize cancer cells [59]. To create particular targeting tools or inherent stimuli-responsive features, pharmaceutical researchers have investigated niosomal activity in anti-cancer treatments for potential use in chemotherapy owing to the adaptability of the constituents. To meet the necessary safety and environmental criteria set for the next 10 years, focus should be placed on the research and development of revolutionary surfactants from alternative renewable raw materials at reasonable rates with improved bioavailability and low toxicity to sustainably provide a wide range of potential applications for cancer treatments. Multi-functional niosomes have also been suggested as a means for creating new opportunities in the pharmaceutical industry. In addition, the use of amphiphilic therapeutic moieties with surface-active qualities allows for crucial advancement in niosomal formulation and synthesis, as the biocompatibility of the vesicle can be elevated [143].

## 12 Conclusion

Curcumin has anti-fungal, anti-bacterial, anti-inflammatory, anti-oxidant, and anti-cancer properties and a high tumor-trapping efficiency, whereas niosomes offer a number of advantages, including a multi-environmental structure that improves curcumin's bioavailability and solubility while simultaneously lowering its toxicity. A unique curcumin-loaded niosome can cure a variety of diseases, primarily cancer. Curcumin niosome technology overcomes the challenges in administering curcumin and permits the effective delivery of medicinal chemicals. The major components of curcumin niosomes are the non-ionic surfactants, cholesterol, charged molecules, and hydration medium. Finally, the curcumin niosome formulation, pH, nature of surfactant, and temperature of the dispersion medium strongly affect the performance of curcumin niosomes. Therefore, curcumin-encapsulated niosomes represent one of the most promising nanomaterials for anti-cancer treatment. The ability of these vesicles to bind to various molecules has been shown to sensitize tumors and provide several cell-killing options for successful cancer eradication *in vitro* and *in vivo*.

### Author contribution statement

All authors listed have significantly contributed to the development and the writing of this article.

## Data availability statement

The authors are unable or have chosen not to specify which data has been used.

## Declaration of competing interest

The authors declare that they have no known competing financial interests or personal relationships that could have appeared to influence the work reported in this paper.
